# Metabolomic credentialing of murine carcinogen-induced urothelial cancer

**DOI:** 10.1038/s41598-021-99746-3

**Published:** 2021-11-11

**Authors:** Hesham Afify, Alia Ghoneum, Sameh Almousa, Ammar Yasser Abdulfattah, Bailey Warren, Kendall Langsten, Daniela Gonzalez, Randy Casals, Manish Bharadwaj, Steven Kridel, Neveen Said

**Affiliations:** 1 Department of Cancer Biology, Medical Center Boulevard, Wake Forest University School of Medicine, Winston-Salem, NC 27157 USA; 2 Department of Urology, Wake Forest University School of Medicine, Winston-Salem, NC 27157 USA; 3 Cell Analysis Division, Agilent Technologies, Inc, Santa Clara, CA 95051 USA; 4 Department of Pathology, Wake Forest University School of Medicine, Winston-Salem, NC 27157 USA; 5 Wake Forest Baptist Comprehensive Cancer Center, Winston-Salem, NC 27157 USA

**Keywords:** Biochemistry, Biological techniques, Cancer, Cell biology, Oncology, Urology

## Abstract

Bladder cancer (BCa) is the most common malignancy of the urinary system with increasing incidence, mortality, and limited treatment options. Therefore, it is imperative to validate preclinical models that faithfully represent BCa cellular, molecular, and metabolic heterogeneity to develop new therapeutics. We performed metabolomic profiling of premalignant and non-muscle invasive bladder cancer (NMIBC) that ensued in the chemical carcinogenesis *N*-butyl-*N*-(4-hydroxybutyl)-nitrosamine (BBN) mouse model. We identified the enriched metabolic signatures that associate with premalignant and NMIBC. We found that enrichment of lipid metabolism is the forerunner of carcinogen-induced premalignant and NMIBC lesions. Cross-species analysis revealed the prognostic value of the enzymes associated with carcinogen-induced enriched metabolic in human disease. To date, this is the first study describing the global metabolomic profiles associated with early premalignant and NMIBC and provide evidence that these metabolomic signatures can be used for prognostication of human disease.

## Introduction

Bladder cancer (BCa) is the fourth most common cancer in men and fifth most common malignancy in the US^1^. Most patients with BCa present with non-muscle invasive bladder cancer (NMIBC) that is treated with bladder preserving therapies. However, despite initial response to therapy, recurrence is high and ~ 50% of patients progress to muscle invasive (MIBC), and metastatic disease with poor prognosis^2^. Patients who present with MIBC or progress to it from NMIBC disease, are treated with radical cystectomy and/or systemic chemo- and radiation therapy, whereas those with locally advanced, recurrent and metastatic BCa have limited treatment options, with a few approved new therapeutics since 2016 with response rate hovering between 15 and 29%. Patients with BCa require repeated treatment courses and life-long surveillance. Therefore, BCa is among the most expensive malignancies to treat from diagnosis to death^2^. Hence, developing more effective anticancer therapeutics relies on the availability of model systems that faithfully recapitulate human BCa not only to understand its pathobiology, but also to identify diagnostic and prognostic markers of disease progression and outcome.

Metabolic programing of cancer cells gained significant attention with the findings that cancer cells undergo contextual programing of their metabolism to fulfill their increasing demands of energy and biomass of the rapidly proliferating cells^3^. Consequently, metabolomic profiling of tumors and associated biological fluids (urine and plasma) are being increasingly reported^4–9^. While these studies highlighted the technical feasibility and the sensitivity of the assays, their integrative analysis with independent transcriptomics shined light on the prognostic utility of the metabolomic signatures associated with disease stage and patient survival. However, these studies were limited by the lack or insufficient numbers of low grade/stage disease as well as appropriate control population^6–9^. Therefore, metabolic credentialing of the model system that faithfully recapitulates the human disease is needed to shine the light on the metabolomic changes in the premalignant and early malignant lesions and would have prognostic value for human disease.

In this respect, the chemical carcinogenesis model in mice using the tobacco metabolite *N*-butyl-*N*-(3-carboxypropyl)-nitrosamine (BBN) offers a surrogate of the human disease as it develops the pathological, genetic, mutational, and transcriptomic features similar to human bladder tumors arising after extensive tobacco use, especially those associated with high-grade MIBC^10–14^. However, metabolomic profiling of BBN-induced carcinogenesis in mice and the metabolic credentialing of this model are still unraveled.

In the present study, we determined the early changes in metabolomics that associate with early premalignant and NMI lesions that developed after 60 and 120 days after BBN carcinogen exposure^11^. We performed comparative analysis using the human metabolome database (HMDB) not only to validate the model, but also to determine the prognostic value of enzymes associated with the significant murine metabolomic signatures with patients’ transcriptomic data from publicly available datasets. Integrated comparative analysis of the key enzymes regulating the enriched metabolites in the BBN murine model and patients’ data revealed that these enzymes are associated with poor disease outcome.

### Significance

Improving our understanding of the pathogenesis of BCa will facilitate the rational development and prioritization of new therapeutic strategies. Our study reports a comprehensive metabolomic profiling of premalignant and NMIBC in an immunocompetent murine model that recapitulates the natural history of the human disease. We show the relevance of the enriched metabolic signatures to human disease outcome. Importantly, we show the dependence of human established BCa cell lines on the key energy generating metabolic pathways not only for ATP production but for survival and invasive phenotypes. These data provide new insights into metabolic vulnerabilities in BCa that represent therapeutic opportunities.

## Results

### Differentially expressed metabolites in premalignant and early malignant urothelial lesions

**Figure 1:**
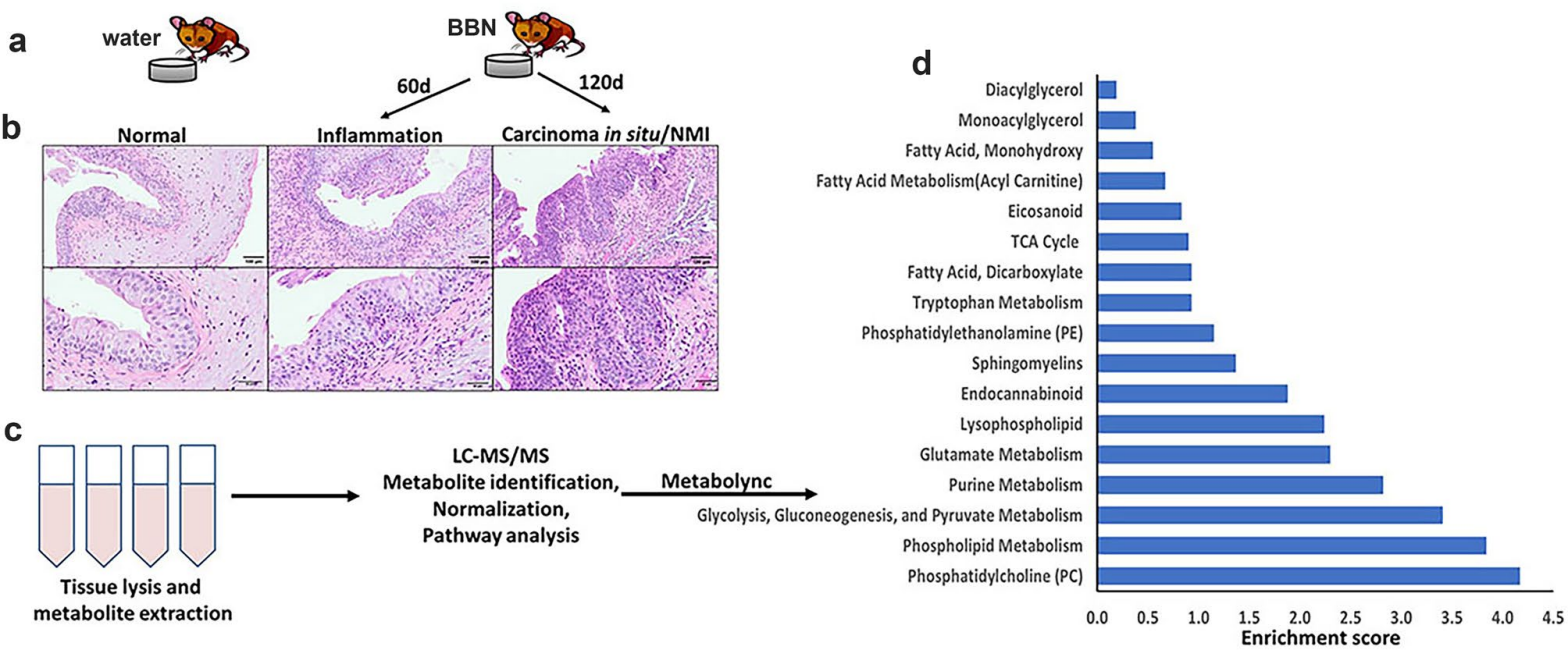
Experimental design: (**a**) Schema of the BBN model. (**b**) H&E images of BBN-induced urothelial lesions at 100x (upper) and 200x (lower) magnification. (**c**,**d**) metabolomic profiling showing enrichment of metabolic pathways.

Consistent with our earlier reports^11^, 90% of mice treated with BBN for 60 days exhibited premalignant changes in the form of atypia, dysplasia, and inflammation, whereas 100% of mice treated with BBN for 120 days exhibited multifocal malignant papillary lesions with CIS and represent NMIBC^11^. Bladder tissue from male mice with bladder pathologic changes were analyzed. Comprehensive metabolomic analysis of bladders from normal and BBN-treated mice detected 568 out of 699 metabolites that were differentially regulated in mice exposed to BBN for 60- and 120-days compared to untreated control littermates (Fig. 1).

### Enrichment of lipid metabolism

**Figure 2:**
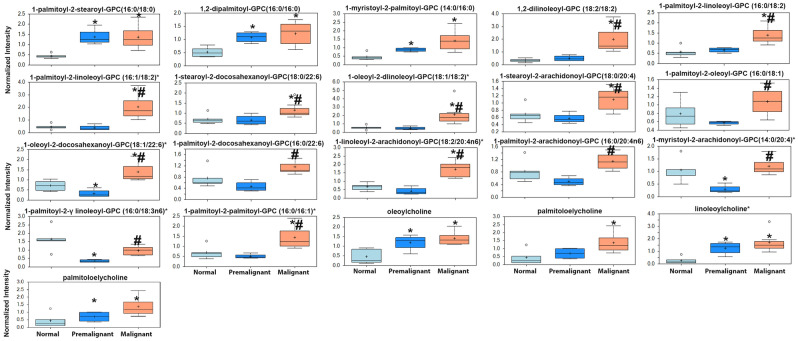
Upregulation of phosphatidyl choline metabolites and choline-conjugated FA. The expression levels of the indicated metabolites involved in phosphatidylcholine pathway *p < 0.05 compared to normal bladders (n = 5). #*p* < 0.05 comparing premalignant lesion (n = 4) to early malignant/NMIBC (n = 7); one-way ANOVA with multiple comparisons.

**Figure 3:**
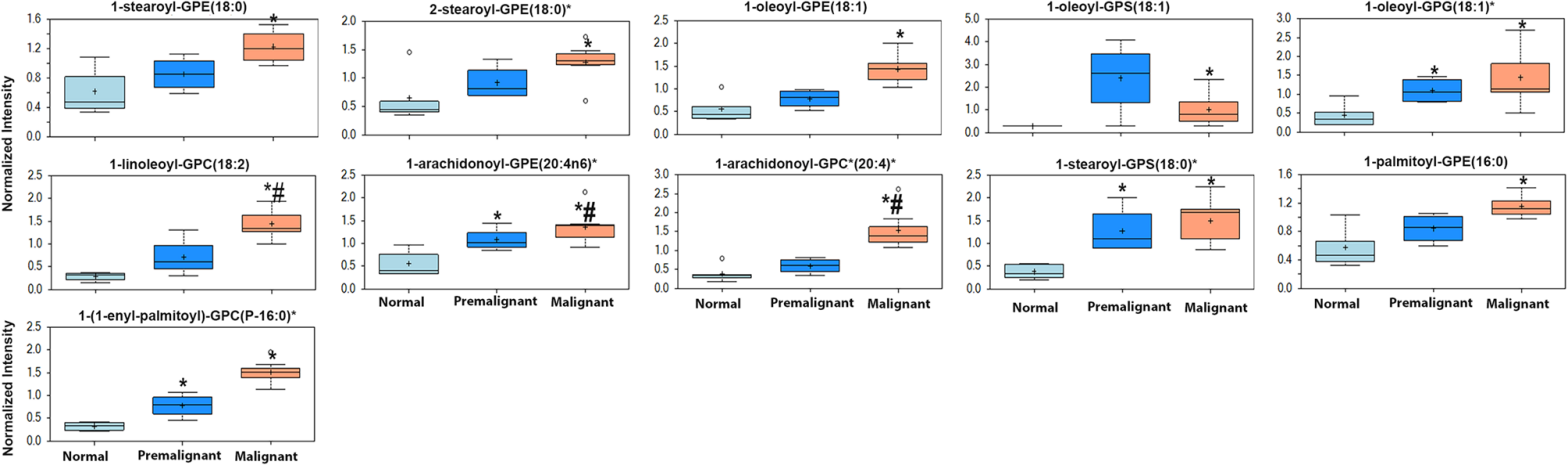
Upregulation of phospholipid and lysophospholipid metabolites: the expression of glycerphosphoethanolamine (GPE), glycerphosphocholine (GPC), glycerophosphoserine (GPS), glycerophosphoglycerol (GPG) and lysophospholipids in normal, premalignant, and early malignant bladder lesions. *p < 0.05 compared to normal bladders (n = 5). #*p* < 0.05 comparing premalignant lesions (n = 4) to early malignant/NMIBC (n = 7); one-way ANOVA with multiple comparisons.

**Figure 4:**
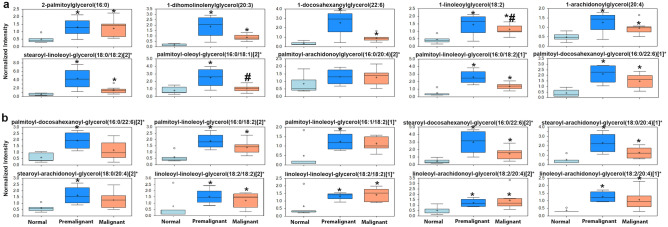
Expression of (**a**) mono-acyl glycerols, and (**b**) di-acylglycerols in premalignant and early malignant lesions. *p < 0.05 compared to normal, and #*p* < 0.05 comparing premalignant and early malignant lesions, one-way ANOVA with multiple comparisons.

**Figure 5:**
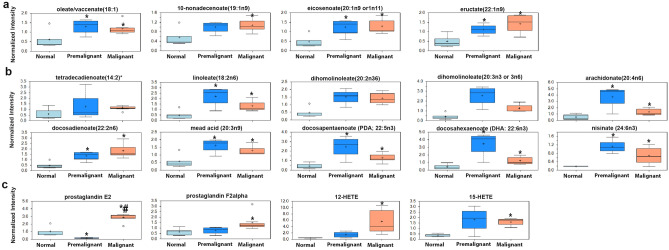
Enrichment of (**a**) long chain fatty acids (LCFA), (**b**) poly-unsaturated fatty acids (PUFA)**,** and (**c**) eicosanoids in normal, premalignant, and early malignant urothelial lesions. *p < 0.05 compared to normal. #*p* < 0.05 comparing premalignant to malignant lesions, one-way ANOVA with multiple comparisons.

Premalignant and early malignant NMIBC BBN-induce murine bladder lesions exhibited significant enrichment of a plethora of lipid metabolites. Pathway enrichment analysis demonstrate that 12 out of 38 enriched metabolite pathways were lipids with 163 out of 492 enriched metabolites in the enriched pathways were lipids or lipid products (Fig. 1d). Phosphatidylcholines (PC) and choline-conjugated FA (Fig. 2) followed by phospholipids and lysophospholipids species as glycerphosphoethanolamine (GPE), glycerphosphocholine (GPC), glycerophosphoserine (GPS), glycerophosphoglycerol (GPG), and lysophospholipids (Figs. 2, 3) exhibited the highest enrichment score with metabolites exhibiting significant increase in premalignant and NMIBC lesions compared to normal bladders. Other lipid species that were significantly enriched include mono- and di-acyl glycerols (Fig. 4). Compared with normal bladders, premalignant and NMIBC exhibited a tendency toward longer acyl chain lengths between 16–24 carbon fatty acids (Fig. 5a), as well as poly-unsaturated fatty acids (PUFA, Fig. 5b).

Consistent with our earlier report^11^, the levels of prostaglandins E2 (PGE2) and PGF2α were significantly upregulated in premalignant and NMI lesions compared to controls (Fig. 5c) suggestive of the increased activity of cyclooxygenase 2 (COX2) enzyme that catalyzes the production of prostaglandins from arachidonic acid/arachidonates^15^. In addition, other pro-inflammatory eicosanoid lipoxygenaseas, 12-Hydroxyeicosatetraenoic acid (12-HETE) and 15-Hydroxyeicosatetraenoic acid (15-HETE), are also upregulated in the premalignant and NMIBC tissues (Fig. 5c). These data suggest that early bladder carcinogenesis is associated with a shift toward de novo lipid metabolism to provide phospholipid species crucial for cell and organellar membrane structure as well as oncogenic signal transduction.

### Clinical relevance of enzymes involved in phospholipid metabolism

Metabolism of phosphatidylcholines (PC), lysophospholipids, and other phospholipid species can be attributed to a series of enzymes including lysophospholipid O-acyltransferases (*LPCATs*), lysophospholipases (*LYPLAs),* phospholipases (*PLAs*) as well as other lipases with phospholipase functions as phospholipase A2 group IIF (*PLA2G2F), patatin-like phospholipase domain containing 3 (PNPLA3), phospholipase B1 (PLB1), lipase C (LIPC),* and *lipase G (LIPG*)^16,17^. Therefore, we determined the transcript levels of this enzymatic machinery in NMIBC patients compared to normal in multiple datasets including TCGA^18^, *GSE13507*^19^, *GSE3167*^20^, and MSKCC^21^ (Supplement Figure 1). We found that *LPCAT1* expression was significantly upregulated in TCGA, *GSE13507,* and *GSE3167,* whereas *LPCAT3* was significantly upregulated in TCGA, but significantly downregulated in *GSE3167* dataset. Consistently, lysophospholipases *LYPLA1* and *LYPLA2* are upregulated in TCGA as well as *GSE3167*. Furthermore, the transcripts of other phospholipases and lysophospholipases *PLA2G2F, PNPLA3, PLB1, LIPC,* and *LIPG* were significantly upregulated in TCGA but they did not exhibit changes in the other datasets (Supplement Figure 1). It is noteworthy that transcripts of some of these enzymes were not detected in some of the available datasets. This may be attributed to different analysis platforms, data processing or normalization. In addition, the discrepancy in transcript expression in different datasets can be attributed to redundancy in enzyme functions, patients’ population, and/or the aforementioned technical variations.

The increased eicosanoids and leukotrienes are consistent with our earlier report^22^ of the positive correlation of the expression of pro-inflammatory eicosanoids with progressive increase in tumor associated macrophage (TAM) infiltration and the levels of pro-inflammatory cytokines and chemokines in the premalignant, malignant and BCa^11^. Consistently, aberrant metabolism of PGE_2_ in BCa has been reported to inhibit the differentiation of recruited myeloid cells and promote accumulation of myeloid-derived suppressor cells within tumor tissue thus promoting tumor immune evasion^23^. However, the expression of *PTGS2, ALOX12,* and *ALOX15* transcripts exhibited inconsistent patterns among TCGA, and the other studies (Supplement Figure 2).

### Dynamic changes in de novo lipogenesis and β-oxidation in premalignant and early malignant lesions

**Figure 6:**
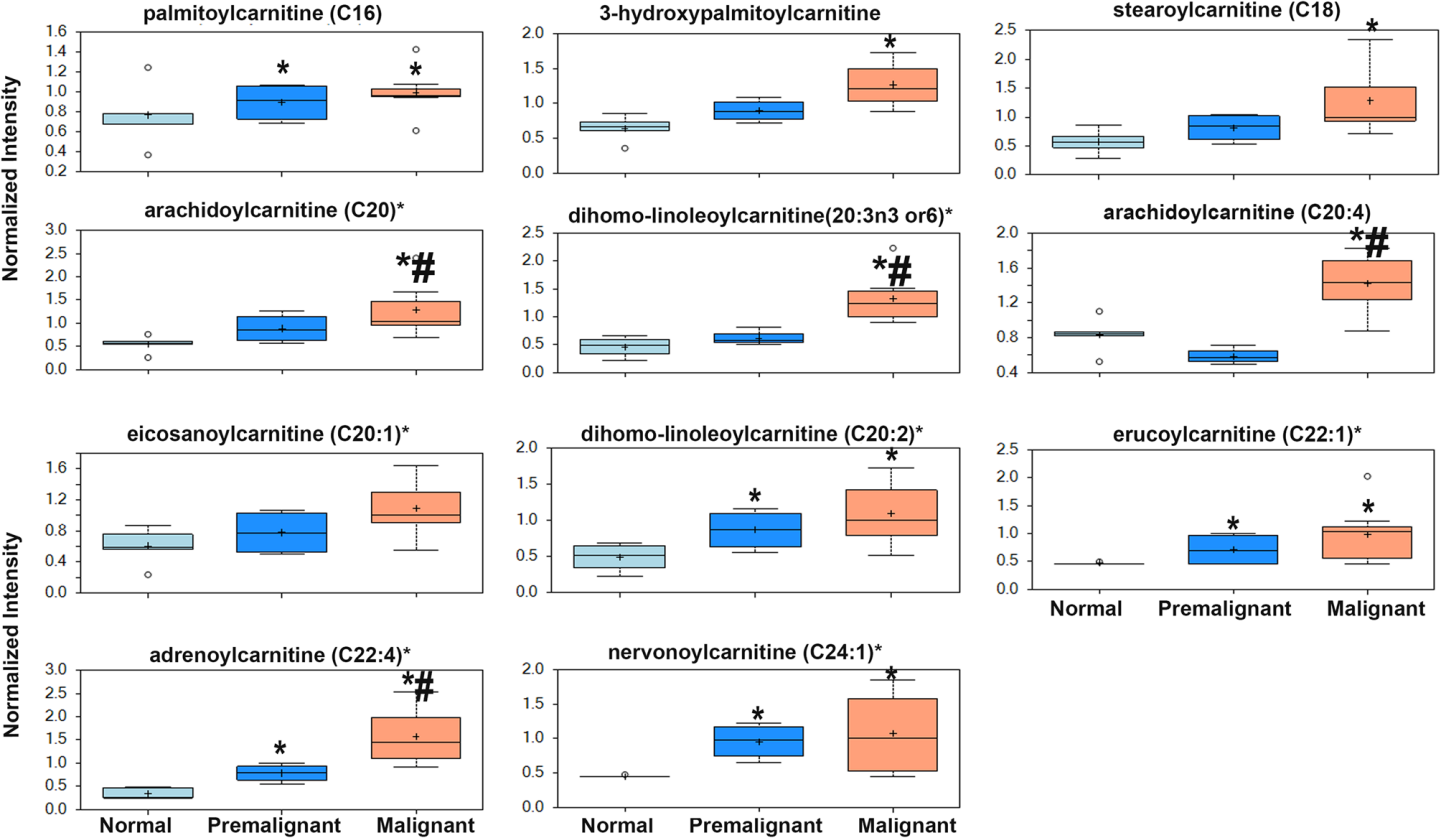
Progressive upregulation of carnitine-conjugated FA in premalignant and early malignant murine bladder lesions. *p < 0.05 compared to normal, and #*p* < 0.05 comparing premalignant to malignant lesions, one-way ANOVA with multiple comparisons.

Premalignant and NMI urothelial lesions exhibited significant enrichment of many species of conjugated long chain FA including long chain acyl carnitines, including dihomo-linoleoylcarnitine (C20:2), dihomo-linolenoylcarnitine (C20:3n3 or 6) and adrenoylcarnitine (C22:4) (Fig. 6), and long-chain glycine- and choline-conjugated fatty acids, mono- and diacyl glycerol, polyunsaturated FAs and long chain fatty acids.

**Figure 7:**
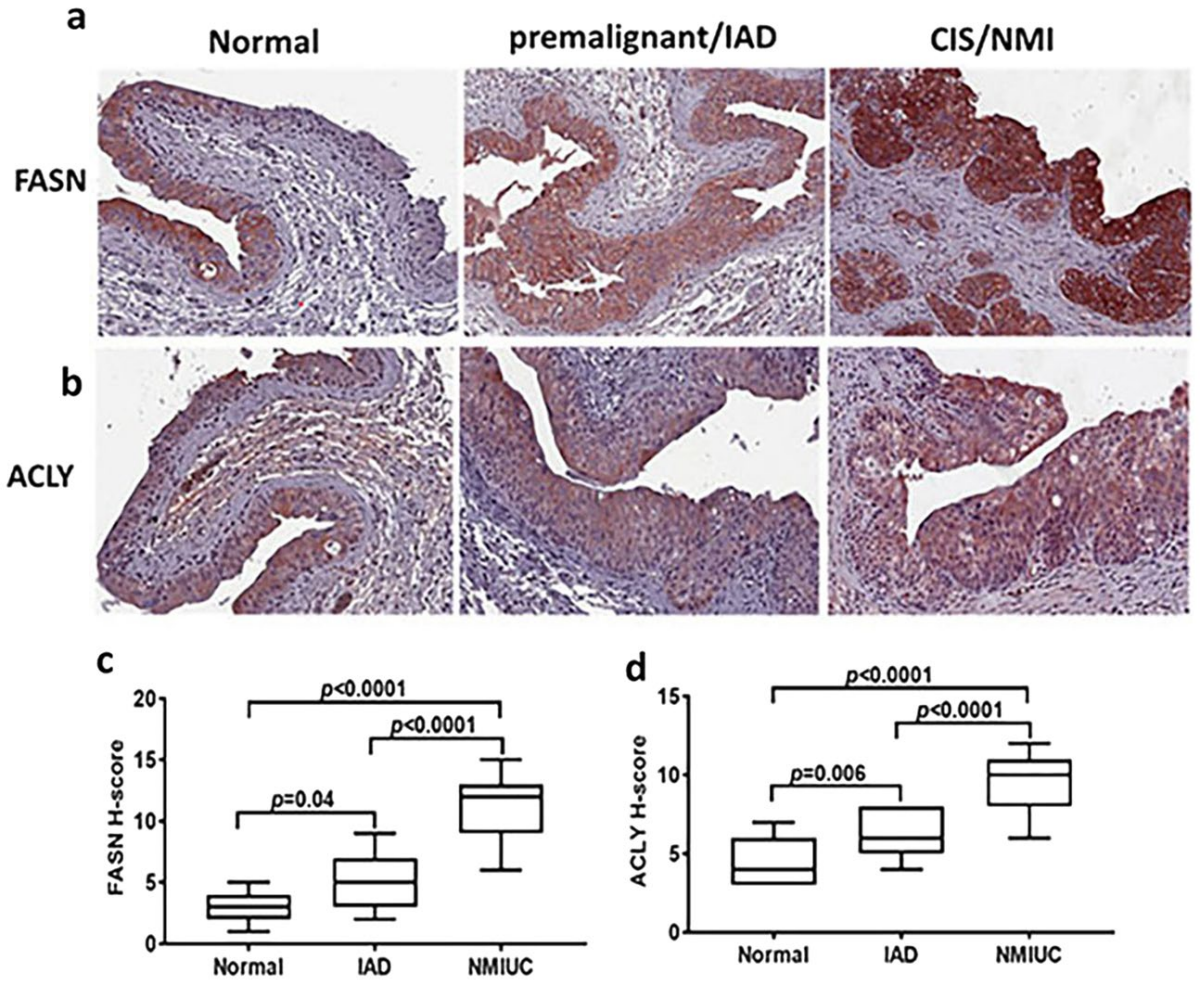
The expression FASN and ACLY proteins in bladder lesions. (**a**,**b**) IHC staining of FASN and ACLY in normal, premalignant and NMI lesions (200 × magnifications). (**c**,**d**) Box plots of the H-scores of the intensity and frequency of the immuno-staining. *p*-values were determined by one-way ANOVA with multiple comparisons.

This increase in the long chain FA and carnitine-conjugated long-chain FA (n18-n24) suggests the activation of FA synthesis and FA β-oxidation in urothelial pre- and early malignant lesions. In support of the metabolic shift towards increased FA synthesis is the significant decrease of citrates concomitant with increased protein expression of ATP citrate lyase ACLY as well as FASN as determined by immunostaining of tissue sections of premalignant and NMI lesions (Fig. 7a–d). Consistently, significant increase of *FASN* and *ACLY* in patients’ tumors was observed in all 4 datasets (Supplement Figure 3a). Acetyl CoA carboxylase A (*ACACA)* was significantly increased in *GSE13507* with a trend though insignificant increase in TCGA, *GSE3167,* and MSKCC datasets. Conversely, *ACACB* transcripts were significantly decreased in TCGA, *GSE13507*, and *GSE3167* datasets, but did not exhibit any change in MSKCC dataset (Supplement Figure 3a). Furthermore, *CPT1A, CPT1B, and CPT2* transcripts exhibited inconsistent expression trends in patients’ tumors compared to normal urothelium (Supplement Figure 3b). Although high expression of *CPT1A* significantly correlated with poor patients’ survival, *CPT1B* transcripts have been reported to be downregulated in high grade bladder tumors and positively associated with patients’ survival^9^. Similar inconsistencies have been observed in the expression of *CPT* enzyme family and their association with patients’ survival in *GSE13507*^19^ (Supplement Figure 3). Analysis of the expression of de novo lipogenesis enzymes in the molecular subtypes of BCa in TCGA data revealed significant upregulation of *FASN* and *ACLY* transcripts in all subtypes compared to normal. Interestingly, FASN transcripts were significantly higher in luminal compared basal squamous subtypes (Supplement Figure 4a). However, *ACLY* transcripts did not exhibit significant difference between luminal and basal subtypes, with significant difference between neuronal and other molecular subtypes (Supplement Figure 4b).

### Effect of targeting fatty acid metabolism on BCa cell lines

**Figure 8:**
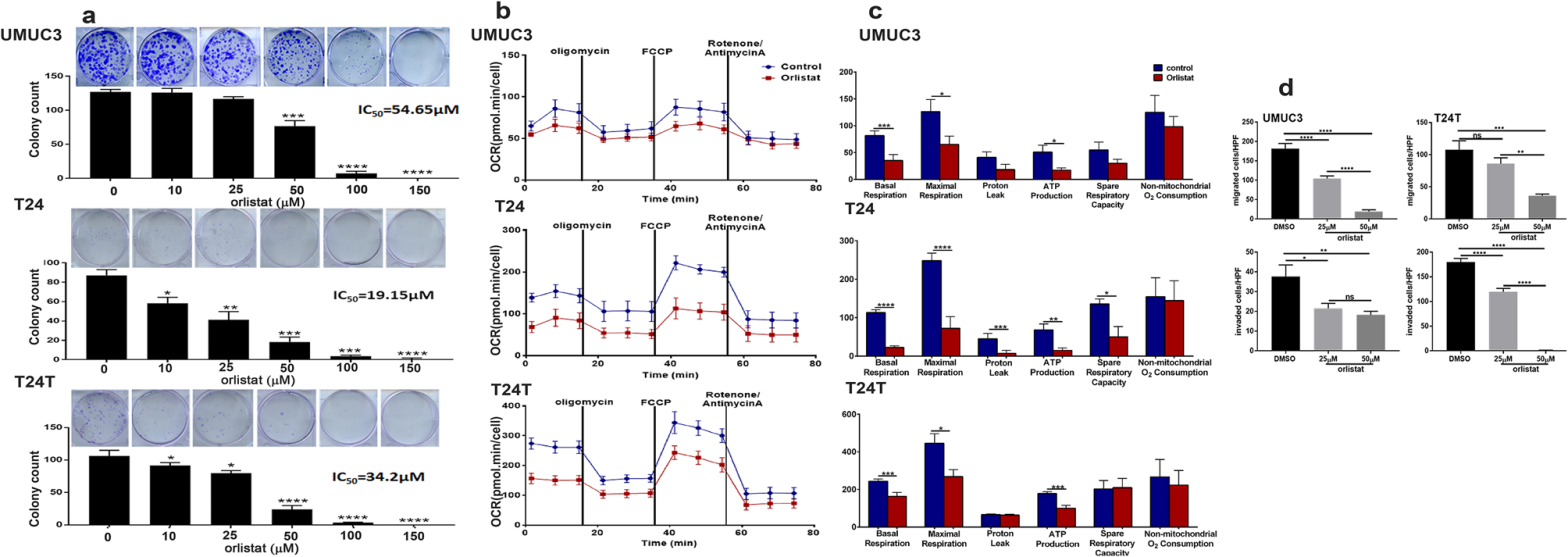
Effect of FASN inhibition by orlistat on BCa cells: (**a**) orlistat exerts dose-dependent inhibition of clonogenic survival of the indicated BCa cell lines. Representative images of colonies > 50 cells after treatment with orlistat. Bar graphs report mean ± SEM of colony counts/well. All experiments were performed in triplicates/experimental condition and were repeated twice. (**b**) Line tracing of the oxygen consumption rate (OCR) of BCa cells treated with 20 µM orlistat or DMSO control. (**c**) Bar graphs report mean ± SEM of indicated measurements (n = 5/experimental condition) repeated twice. (**d**) Orlistat exerts dose-dependent inhibition of UMUC3 and T24T migration and matrix invasion. Bars report mean ± SEM of number of migrating and matrix-invading cells/high power field (HPF). *p < 0.05, **p < 0.01, ***p < 0.001, ****p < 0–0001 and ns, not significant. One-way ANOVA with multiple comparisons (**a**) and Student’s t-test (**c**,**d**)**.**

We next sought to determine the requirement of fatty acid synthesis (de novo lipogenesis) and β-oxidation for the malignant phenotype and ATP production of established BCa cell lines. We found that orlistat, a lipase inhibitor with thio-esterase FASN inhibitor domain^24,25^ exerted dose-dependent inhibition of survival of BCa cell lines UMUC3, T24, and T24T with IC_50_ of 54.65 µM, 19.15 µM and 34.2 µM, respectively (Fig. 8a). Similarly, a specific FASN inhibitor TVB3664 exerted similar inhibitory effect of BCa cell clonogenic survival with IC_50_ of 23.5 µM and 11.5 µM, for T24 and T24T, respectively (Supplement Figure 5). Real time monitoring of mitochondrial bioenergetics determined by O_2_ consumption rate (OCR) of BCa cell lines treated with orlistat revealed that orIistat significantly inhibited basal and maximal respiration, and ATP production in UMUC3, T24, and T24T cell lines (Fig. 8b,c). Furthermore, orlistat (Fig. 8d) and TVB3664 (Supplement Figure 5) significantly inhibited UMUC3 and T24T invasiveness. These findings indicate that targeting de novo lipid synthesis inhibited BCa malignant phenotype and metabolic programing. It is noteworthy that the invasive/metastatic T24T cells exhibited significantly higher basal mitochondrial respiration compared to their isogenic non-invasive T24 cells suggesting higher metabolic demands and energy requirements of the invasive cells. In addition, orlistat and TVB3664 exerted a modest insignificant effect on clonogenic survival, migration, and invasiveness on RT4 BCa cell line when used at the same concentration range and experimental duration as used with the other cell lines (not shown).

To determine the dependence of BCa cells on FAO, we examined the effect of CPT1a specific inhibitor, etomoxir on the malignant phenotype of BCa cells. We found that etomoxir inhibited the 3 BCa cell survival with IC_50_ of 83.5 µM, 110.9 µM, and 60.8 µM for UMUC3, T24, and T24T, respectively (Supplement Figure 6a). Etomoxir significantly inhibited basal and maximal respiration, spare respiratory capacity, and non-mitochondrial O_2_ consumption in the 3 cell lines with a trend through insignificant decrease in ATP production (Supplement Figure 6b-c). Furthermore, etomoxir inhibited T24T but not UMUC3 cell invasiveness (Supplement Figure 6d). These findings suggest that while inhibition of FAO in BCa cells is sufficient to inhibit their mitochondrial bioenergetics, it is not sufficient to inhibit their survival and invasiveness.

### Enrichment of glucose and glycolysis pathways

**Figure 9:**
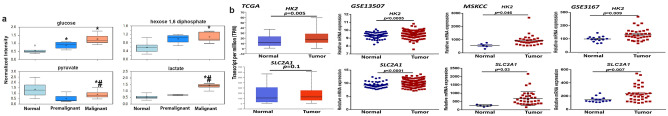
Glycolysis in premalignant and early malignant bladder lesions: (**a**) The expression of glucose and glycolysis intermediate and end metabolites. *p < 0.05 compared to normal. #*p* < 0.05 comparing premalignant to malignant lesions. (**b**) Transcript expression of *HK2* and *SLC2A1* (glucose transporter 1) in NMI tumors compared to normal urothelium in TCGA, *GSE13507*, MSKCC, and *GSE3167. p*-values were determined by one way ANOVA (**a**), and Student’s *t*-test (**b**).

Comprehensive metabolomic profiling of murine bladder lesions also revealed enrichment of glucose metabolism and glycolysis (Fig. 1) evidenced by significant increase in glucose as well as hexose diphosphates (fructose 1.6-diphosphate, glucose 1,6-diphosphate and myoinositol 1,6-diphosphate). In addition, glycolytic end-product lactate was also significantly elevated (Fig. 9a). These data suggest increased glucose uptake, with enhanced glycolysis. Consistently, the transcripts of glucose transporter 1 (*SLC2A1*) were significantly increased in *GSE13507*, MSKCC, and *GSE3167* datasets but not TCGA, whereas those of hexokinase-2 (*HK2*) were significantly increased in the 4 datasets (Fig. 9b). To further determine the dependence of BCa cell lines on glycolysis for their malignant phenotype, we treated BCa cell lines UMUC3, T24, and T24T with 2-deoxyglycose (2-DG), an allosteric inhibitor of HK2 and found that it exerted a dose-dependent inhibition of clonogenic survival of BCa cells with IC_50_ of 35.51 µM, 412.5 µM, and 18.08 µM for UMUC3, T24, and T24T, respectively (Supplement Figure 7a). 2-DG also inhibited UMUC3 and T24T invasiveness (Supplement Figure 7b).

### Enrichment of TCA cycle in premalignant and early malignant bladder lesions

**Figure 10:**
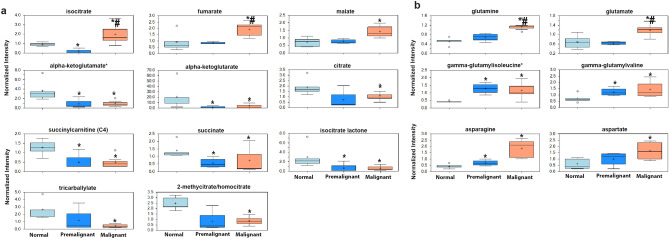
TCA cycle metabolites in premalignant and malignant murine lesions: Box plots show the expression of (**a**) TCA cycle metabolites. (**b**) glutamine, glutamates, asparagine and aspartate in premalignant and early malignant bladder lesions. *p < 0.05 compared to normal bladders (n = 5). #*p* < 0.05 comparing premalignant lesion (n = 4) to early malignant/NMIBC (n = 7); one-way ANOVA with multiple comparisons.

The levels of citrate, succinate, and α-ketoglutarate exhibited significant decrease in premalignant and early malignant bladders compared to normal controls. However, TCA end products fumarate and malate were significantly increased in premalignant and early malignant urothelial lesions compared to controls (Fig. 10a). In addition, other TCA intermediate metabolites also exhibited significant decrease as α-ketoglutaramate, succinyl carnitine, tricarballylate, and isocitric lactone (Fig. 10a). Interestingly, the levels of glutamine, glutamate, gamma glutamyl isoleucine, and gamma glutamyl valine were significantly increased in premalignant and NMIBC lesions compared to controls (Fig. 10b). The changes in glutamine/glutamate metabolites suggest either the accelerated conversion of α-ketoglutarate and α-ketoglutarate to glutamate for anaplerosis, nucleotide biosynthesis or glutathione synthesis for redox homeostasis^26^. These data are consistent with earlier reports of the increase of glutamine and glutamate derivatives in patients’ bladder tumors to provide energy for the anaplerotic effect observed with increased late TCA intermediates^8^. To further determine the dependance of BCa cells on TCA cycle for malignant phenotype and energy production, we used CPI-613® (devimistat) that specifically targets the mitochondrial TCA cycle and is currently FDA approved for hematologic malignancies^27–31^. We found that CPI-613 inhibited clonogenic survival of BCa cell lines with IC_50_ of 53.15 µM, 76.07 µM, and 154.5 µM for UMUC3, T24, and T24T, respectively (Supplement Figure 8a) as well as their matrix invasiveness (Supplement Figure 8b).

### Tryptophan metabolism

Premalignant and NMI lesions exhibited significant elevation in tryptophan and kynurenine (Supplement Figure 9a) concomitant with decrease in their metabolic degradation products indolacetate, 5 hydroxyindolacetate, and kynurenate (Supplement Figure 9a). Thus, we determined the transcript levels of the enzymes involved in their dynamic metabolism and found that significant upregulation of enzymes that catalyze the conversion of tryptophan to kynurenine tryptophan-2,3-dioxygenase 2 (*TDO2*), and indolamine-2,3-dioxygenase (*IDO1*) (Supplement Figure 9b). Both are produced in the tumor microenvironment to induce anti-tumor immune responses leading to immune tolerance and inflammation^32,33^. Consistently, kynureninase (*KYNU*) that catalyzes the conversion of kynurenine into kynurenate and anthranilic acid is significantly downregulated in patients’ tumors in TCGA dataset (Supplement Figure 9b). This is further evidenced in our earlier report of increased tumor associated macrophages^34^, as well as the concomitant significant increase in inflammatory mediators, eicosanoids PGE2 and PGF2α.

### Significant enrichment of purine metabolites

Premalignant and early malignant lesions exhibit significant stepwise increase of purine nucleotides specifically adenine, adenosine, adenosine monophosphate (AMP), adenosine 2' monophosphate (2'-AMP), adenosine 3'-monophosphate (3’-AMP), 3'-5-adenylyadenosine, 3’-5’-adenylyluridine. Whereas ADP is decreased, suggestive of rapid turnover of purines for both energy and nucleotide synthesis (Supplement Figure 10).

## Discussion

Most metabolomics studies in BCs have focused on the comparison between NMIBC and MIBC, highlighting the importance of several metabolites involved in pathways related to energy production, and BCa aggressiveness^4,5^. However, the predictive, diagnostic, or prognostic utility of metabolomics is still limited due to lack of a model system that faithfully recapitulates the natural history of the human disease. In the present study, we used the well-established BBN-chemical carcinogenesis murine model to report the early metabolic changes that ensue in murine bladders after carcinogen exposure, and compared these changes in premalignant, and early NMIBC lesions. We also determined the expression of the enzymes that catalyze the metabolic pathways enriched in murine model with their expression in patients’ specimens from public datasets that included normal and NMIUC specimens. We further determined the effect of inhibition of the major energy generating pathways on survival, invasiveness, and ATP production of established BCa cell lines.

Herein, we report that the earliest changes that occur in preneoplastic lesions are mainly enrichment of lipid metabolism specifically phospholipids moieties crucial for plasma membrane structure and hence dictate the oncogenic signaling pathways^35–37^. Changes in the major constituents of the plasma membranes represent modifications of phosphatidic acid conjugated with inositol (phosphatidylinositol), glycerol (phosphatidylglycerol, PG), serine (phosphatidylserine, PS), ethanolamine (phosphatidylethanolamine, PE), choline (phosphatidylcholine, PC), and glycerophosphocholine (GPC)^38,39^; all impact numerous cellular processes, including cell growth, proliferation, differentiation, and motility^40^. The dynamic interconversion of the various phospholipids is catalyzed by lysophosphatidylcholine acyltransferases *(LPCATs)* localized in cell and organelle membranes where they catalyze the transfer of the fatty acyl chains from fatty acyl-CoA to 1-acyl lysophospholipid to form various classes of phospholipids^16,17^, leading to the dynamic interconversion of phospholipid species^16,17,41^ as indicated in Figs. 2, 3, 4 and 5. In addition, phosphatidylcholine-specific phospholipases co-localize with membrane receptor tyrosine kinases where they reciprocally lead to overexpression and activation of each other as well as downstream oncogenic signaling^42^. The dynamic changes in the phospholipid content of cell membranes not only determine cell motility^35,37,39,43^, but also provide substrates and secondary messengers for the phosphatidyl inositol 3 kinase (PI3K) pathway that links oncogenic signaling to downstream survival, invasive and metabolic programing and is hyperactivated BCa^44^. Consistently, many phospholipid species were reported to be altered in the urine of BCa patients suggesting their association with high tumor proliferation rate with increased cell membrane remodeling^45,46^. Therefore, it was not surprising to find variabilities in the expression of transcripts of *LPCATs* among the different patients’ datasets reported herein. Furthermore, the increased activity of phopholipases was associated with increased levels of arachidonate and leukotrienes; both can be attributed to the release of FFAs from cell membrane phospholipids within or around cancer cells^45,46^, and significantly contribute to the inflammatory microenvironment that precedes and drives BCa progression and metastasis^11,22,47^.

The increased lipid moieties in murine lesions also suggest that carcinogen exposure upregulates de novo lipogenesis in the early premalignant lesions. Indeed, that was the case as we found significant upregulation of the protein expression of enzymes catalyzing de novo lipogenesis *ACLY* and *FASN* in premalignant lesions and progressed in early malignant NMI murine lesions. Interestingly both FASN and ACLY were significantly upregulated in the four BCa patients’ datasets that we interrogated and the expression of *FASN* positively correlated with tumor aggressiveness, advanced grade and stage, and poor prognosis^48,49^. The concomitant significant decrease in citrates suggests metabolic shift towards de novo lipogenesis. These were further confirmed by pharmacologic inhibition of FASN and ACLY by orlistat and TVB3664 (inhibitors of FASN) as well as NDI-091143 (an inhibitor of ACLY) on BCa cell clonogenic survival, and invasiveness. Importantly, inhibition of FASN by orlistat inhibited ATP production and mitochondrial basal and maximal respiration as determined by oxygen consumption rates (OCR).

Premalignant and early malignant murine bladder lesions exhibited a significant increase in carnitine-conjugated FAs, however, carnitine-conjugating enzymes, were not changed at the protein level (not shown). While significant changes in carnitine species have been noted in BCa patients^50,51^, CPTs’ transcript expression exhibited inconsistent trends in the four BCa datasets. In addition, multi-OMICs analysis of TCGA data revealed that *CPT1B* was associated with a better disease outcome in patients as well as preclinical models^9^. Phenotypically, our data revealed that inhibition of CPT1 by etomoxir exerted a modest inhibitory effect on BCa cells, survival and invasiveness. Etomoxir did not exert a significant inhibitory effect on ATP production despite significant inhibition of both basal and maximal mitochondrial respiration. These data suggest redundancy in effects of CPTs on β-oxidation as a source of energy in BCa.

Our data also revealed the accelerated glycolysis evidenced by significant increase in glucose, glycolysis intermediates as well as the end-product lactate, consistent with previous studies showing increased production of lactate in both BCa tissue and urine indicating higher glycolytic rates^52^. This is further supported by the significant upregulation of *HK2* in the four BCa datasets, and the inhibitory effect of 2-DG on clonogenic survival and invasiveness of established BCa cell lines. In addition, the increased lactate levels support an acidic hypoxic tumor microenvironment as an adaptive mechanism in the premalignant lesions. Such acidic conditions also play an important role in cancer aggressiveness and metastasis with subsequent suppression of the immune system^53,54^. In further support of the immunosuppressive BCa tumor microenvironment is the significant increase in tryptophan and kynurenine in premalignant and NMI lesions after BBN carcinogen exposure. Both have been reported as oncometabolites that inhibit natural killer (NK) cell and T-lymphocytes and promote cancer cell survival and immune evasion^32,33,55–57^. Consistently, transcripts of the enzymes involved in the kynurenine pathway as IDO1 and TDO2 are significantly increased in patients with NMIBC compared to normal bladder tissues.

Premalignant and early malignant murine bladder lesions exhibited an accelerated TCA profile evidenced by significant decrease in pyruvate and citrate concomitant with an increase in fumarate and malate. The decreased level of citrates also suggests metabolic shift towards lipid metabolism. In addition, the significant increase in glutamine and glutamates, concomitant with decrease succinate and α-ketoglutarate suggest enhanced anaplerosis to replenish carbon in TCA cycle by glutamine for cellular bioenergetic, and biosynthetic needs as well as redox homeostasis^58^. In further support of the dependence of BCa cells on TCA cycle, CPI-613 (devimistat)^59^, an inhibitor of mitochondrial pyruvate dehydrogenase (PDH) and alpha-ketoglutarate dehydrogenase (α-KGDH) involved in the TCA cycle, significantly inhibited BCa cell survival and invasiveness. Furthermore, the progressive significant increase in gamma-glutamyl-conjugated branched chain amino acids isoleucine and valine suggest enhanced gamma-glutamyl pathway involved in glutathione and xenobiotic metabolism^60,61^. While the transcripts of γ-glutamyl transferases (GGTs) did not exhibit consistent changes in BCa datasets, elevated serum GGT was reported as an independent adverse prognostic factor in BCa patients^62,63^.

Finally, purine metabolite AMP exhibited significant stepwise increase in premalignant and early malignant lesions indicating the rapid utilization for generation of ATP and rapid turnover of ATP in the carcinogenesis process.

In summary, our data show for the first time that BBN carcinogen induces changes in the metabolic landscape in premalignant lesions and early malignant NMI bladder lesions. Comparative cross-species analysis of the rate limiting enzymes of the key enriched signaling pathways, revealed consistent significant upregulation of enzymes regulating de novo lipogenesis *FASN* and *ACLY* in murine bladder tissues and in four independent BCa patients’ datasets. Both glycolysis and TCA cycle metabolites were enriched in BBN-induced premalignant and NMI lesions indicating metabolic plasticity to generate energy, nucleotides and biomass. Importantly, pharmacologic targeting of the rate limiting enzymes of the enriched metabolic pathways identified metabolic dependence of BCa cells, exemplified in invasive cell line T24T exhibiting higher mitochondrial bioenergetics profile and lower IC_50_ of the inhibitory drugs compared to their non-invasive isogenic T24 cells, highlighting the metabolic vulnerability that can be exploited as therapeutic target.

## Material and methods

### Mice and chemical carcinogenesis model

All animal procedures were approved by the IACUC of Wake Forest University School of Medicine (IACUC protocol #A15-145 and A18-145). All methods were performed in accordance with the relevant guidelines and regulations at Wake Forest University School of Medicine. Six-week-old male C57BL/6 J were purchased from Jackson Labs (Bar Harbor, ME). Mice were fed 0.05% BBN in drinking water^11^ for 60 and 120 days after which they were euthanized by isoflurane inhalation and cervical dislocation. All experimental procedures and results are performed and reported in accordance with the ARRIVE guidelines (https://arriveguidelines.org). Bladders were dissected and one half was formalin-fixed and paraffin embedded for H&E and IHC and the other half was snap–frozen in liquid nitrogen and stored at –80 °C till they were processed for metabolomic profiling.

### Metabolomic profiling

Extraction and profiling of metabolites from murine bladders were done using Metabolon Platform (Metabolon Inc., Raleigh, NC) as previously described^64–66^. Extraction and profiling of metabolites from bladders from BBN-treated mice and untreated controls were conducted at Metabolon Inc. as previously described^65,66^. Following peak identification and QC processing, metabolite values were normalized by tissue weight for each sample and median scaling of each metabolite across all samples and imputation of each metabolite by the minimum observed value of that compound were performed^65,66^. Pathway enrichment analysis was conducted using MetaboLync webtool (Metabolon Inc).

### Cell lines

All cell culture reagents were from Invitrogen. UMUC3 (CRL-1749™) and T24 (ATCC® HTB-4™) cells lines were obtained from and maintained as recommended by ATCC. UMUC3 was originally isolated from pT2-4 tumor of a male patient and is tumorigenic in xenografts in athymic nude mice. UMUC3 exhibit characteristics of basal BCa subtype^67^. T24 cell line is originally isolated from a high grade (G3), PTa tumor from female patient and is non-tumorigenic in xenografts in athymic nude mice; however, T24 exhibit characteristics of basal BCa subtype^67–70^. T24T is an isogenic invasive and metastatic cell variant of T24 and were earlier described^11,22,47,67,70,71^. T24T were authenticated by Short Tandem Repeat (STR) analysis at Genetica Cell Line Testing labs (LabCorps, Burlington, NC) and found to bear 96.30% identity and 92% match to T24 (ATCC® HTB-4™) cells.

Cell lines were used within 5 passages after retrieving from cryo-storage.

### Transcriptomics data

The Cancer Genome Atlas (TCGA) data were analyzed using publicly available web tools UALCAN (http://ualcan.path.uab.edu/)^18^. *GSE13507*^19^ and *GSE3167*^20^ data that comprised normal and NMIBC patient specimens were downloaded from Gene Expression Omnibus (GEO). Memorial Sloan Kettering Cancer Center (MSKCC) cohort data were downloaded from the supplementary material^21^. All data were analyzed using MS-Excel, GraphPad Prism 7.0 (San Diego, CA) and R-Stata software (College Station, TX).

### Drugs and chemicals

Etomoxir, orlistat, TVB3664 and NDI were purchased from Selleck Chemicals (Houston, TX). 2-deoxyglucose (2-DG), was purchased from Sigma Aldrich (St. Louis, MO). CPI-613 was a kind gift from Dr. Tim Pardee, MD at Wake Forest University Health Sciences. Unless otherwise stated, all chemicals were analytical grade and were purchased from Sigma or Thermo Fisher (Fair Lawn, NJ).

### Clonogenic survival

BCa cells were plated in wells of 6-well plates (Corning, ThermoFisher) at a density of 500 cells/ well, and allowed to attach for 16 h, before being treated with the metabolic inhibitors, orlistat, etomoxir, orlistat, TVB3664, NDI091143, 2-DG, and CPI-613, at concentrations indicated in figures and figure legends for further 10–14 days. Formation of cell colonies > 50 cells/colony was confirmed by examination under inverted microscope. Plates were then fixed with 4% paraformaldehyde and were stained with crystal violet (Fisher Scientific). Cell colonies with > 50 cells were counted using eCount™ Handheld Colony Counter (Sigma) and colony counting tool in Image J. All experiments were done in triplicates and were repeated 3 times with reproducible results. The concentration of each inhibitor that inhibits colony formation by 50% (IC_50_) is calculated from the dose response of each drug after log_10_ transformation of the concentration followed by non-linear fitting of normalized transformed values using GraphPad Prism.

### Trans-well migration and invasion assays

BCa cells 1 × 10^5^/100 µl serum-free medium (SFM) were added on top of uncoated (for migration assay) and matrigel-coated (for invasion assay) 8 µm-pore trans-well inserts (Corning, ThermoFisher) and the appropriate complete growth media for each cell line was included in the bottom chamber^11,22,47,72^. BCa cells were incubated at 37 °C for 6-8 h with the metabolic inhibitors at the concentrations described in the figure legends; after which cells in the top chambers were scraped by Q-tips, and inserts were fixed, and stained with Hemacolor 3 (ThermoFisher)^11,22,47,72^. Migrated/invaded cells to the under surface of the inserts were counted in 5 random fields using 20 × magnification using Evos Imaging system (ThermoFisher)^11,22,47,72^.

### Bioenergetics mito-stress assays

Mitochondrial function was assessed using the Agilent Seahorse XFp (8 wells) and XF (24 wells) Extracellular Flux Analyzer (Agilent, Santa Clara, CA, USA). Cells were plated at 50,000 cells/well in DMEM with 10% FBS in 8- or 24-well microplates (3–5 wells/experimental condition with one blank well in each row). Cells were then treated with orlistat (50 µM), or etomoxir (50 µM) for 18 h. Prior to starting the assay, cells were washed, media and inhibitors (orlistat and etomoxir) removed, and plates were incubated in Seahorse assay medium (Agilent Seahorse XF DMEM Medium pH 7.4 #103,575–100), and supplemented with 1 mM pyruvate, 2 mM glutamine and 10 mM glucose in a 37 °C incubator without CO_2_ for 60 min. Plates were then incubated in the Seahorse machine and the following drugs were sequentially injected oligomycin (1 µM), an ATP synthase inhibitor, fluoro-carbonyl cyanide phenylhydrazone (FCCP, 1 µM), an ionophore which shuttles H^+^ ions, and rotenone/antimycin A (0.5 µM), which inhibit complex I and III, respectively as indicated in the line graphs in Fig. 8 and Supplement Figure 6. All parameters were measured in units of oxygen consumption rate (OCR, pmol O_2_/min), namely basal respiration, ATP production-coupled respiration, maximal and reserve capacities, and non-mitochondrial respiration^73^. Data were normalized to the number of viable cells determined by trypan blue exclusion and cell counting at the end of each experiment. Data were calculated, and graphs were plotted using Agilent Seahorse Wave Desktop software and report generator as per manufacturer’s instructions^73^, MS Excel and GraphPad Prism (San Diego, CA)^64,73^.

### Immunohistochemistry (IHC)

Formalin-fixed paraffin embedded bladder sections (4-6 µm thick) were deparaffinized in xylene and descending grades of ethanol. Antigen retrieval was performed by boiling slides in citrate buffer (pH 8.0) for 15 min. Endogenous peroxidase was blocked by incubating slides in 3% H_2_O_2_ in PBS, followed by incubated with rabbit monoclonal antibodies against FASN (clone C20G5, #3180, Cell Signaling Technologies, Danvers, MA) and ACLY (clone EP704Y, #ab40793, Abcam, Waltham, MA) at concentration of 1:50 in blocking buffer (PBST-2% normal horse serum, Vector Labs, Burlingame, CA). After which sections were developed with horseradish peroxidase (HRP)-conjugated secondary antibodies using VECTASTAIN® Universal Quick Peroxidase (HRP) Kit, Peroxidase and ImmPACT® DAB Substrate. Frequency and intensity of immunostaining and H-scores were determined as earlier described^11,47^.

## Supplementary Information

Supplementary Figures.
