# Supplementary material for: Metabolomic credentialing of murine carcinogen-induced urothelial cancer
Source: Sci Rep. 2021 Nov 11;11:22085. doi: 10.1038/s41598-021-99746-3 (PMC8585868; doi:10.1038/s41598-021-99746-3)
Supplement: Supplementary file 1 — Supplementary Figures. [file 41598_2021_99746_MOESM1_ESM.pdf]

S1

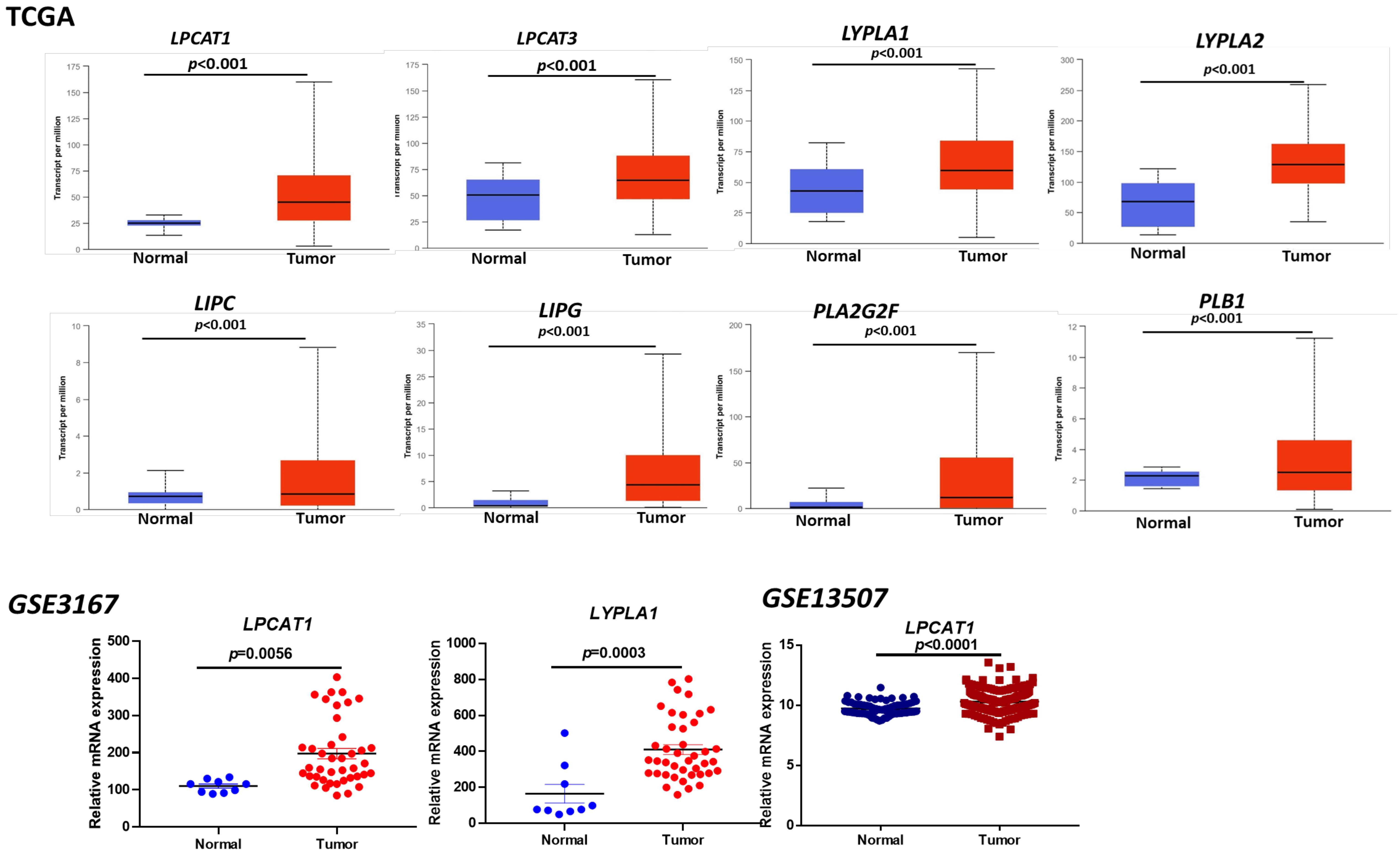
TCGA
LPCAT1
p<0.001
LPCAT3
p<0.001
LYPLA1
p<0.001
LYPLA2
p<0.001
LIPC
p<0.001
LIPG
p<0.001
PLA2G2F
p<0.001
PLB1
p<0.001
Transcript per million
Normal
Tumor
GSE3167
LPCAT1
p=0.0056
LYPLA1
p=0.0003
GSE13507
LPCAT1
p<0.0001
Relative mRNA expression
Normal
Tumor

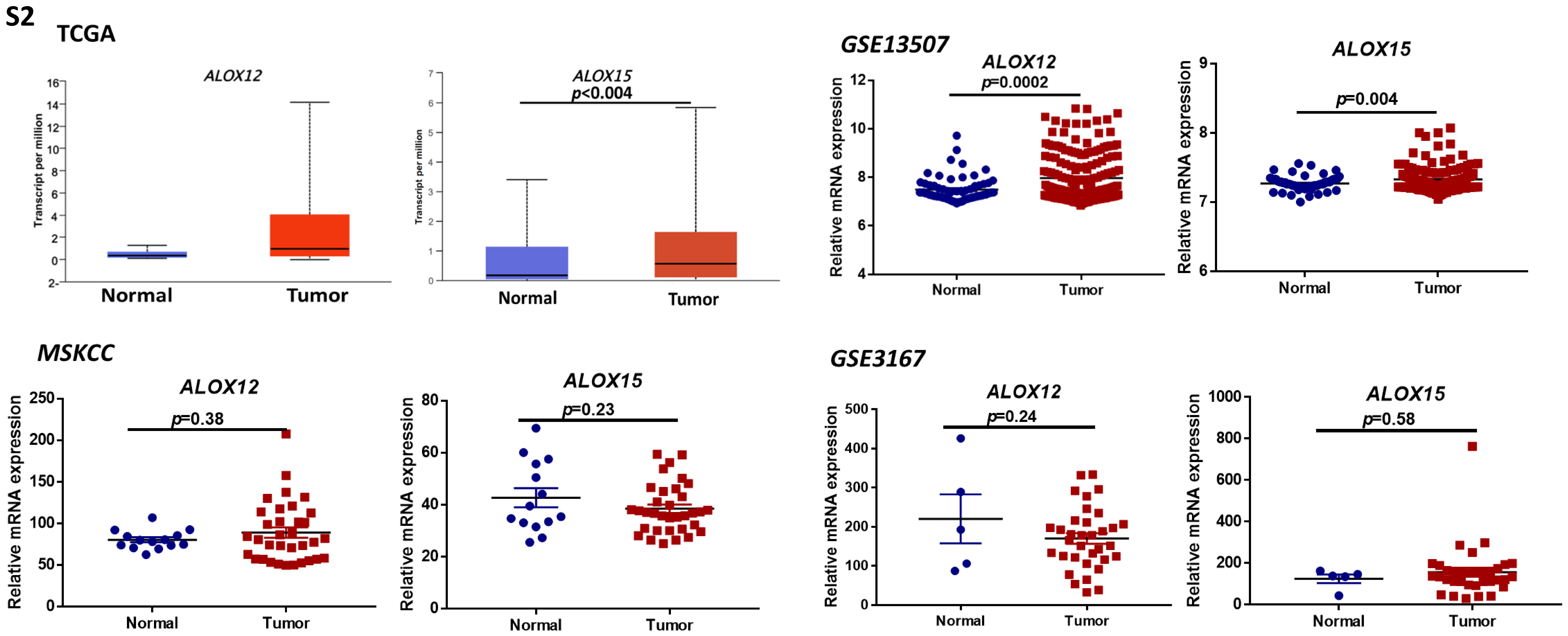
S2
TCGA
ALOX12
Transcript per million
Normal
Tumor
ALOX15
p<0.004
GSE13507
ALOX12
p=0.0002
Relative mRNA expression
ALOX15
p=0.004
MSKCC
ALOX12
p=0.38
ALOX15
p=0.23
GSE3167
ALOX12
p=0.24
ALOX15
p=0.58

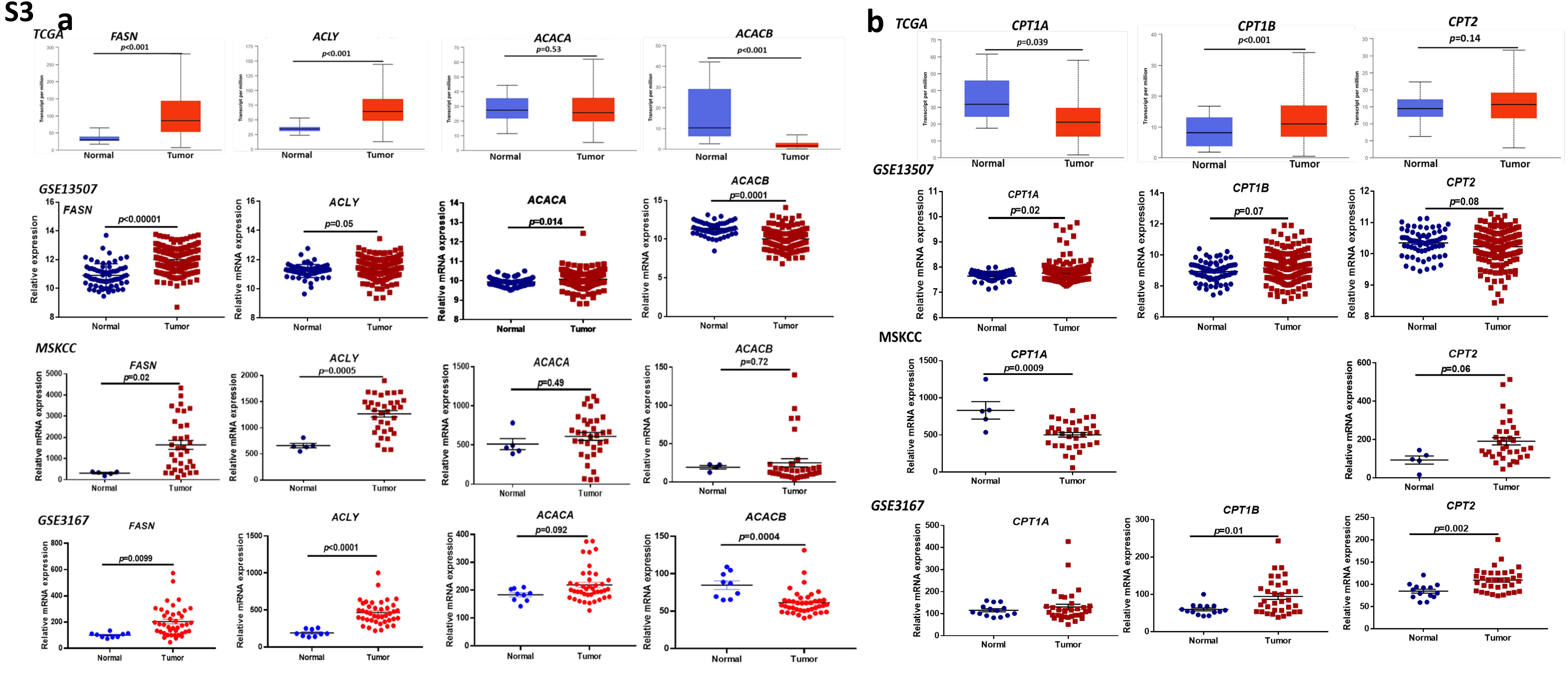
S3
a
b
TCGA
FASN
ACLY
ACACA
ACACB
CPT1A
CPT1B
CPT2
GSE13507
MSKCC
GSE3167
Normal
Tumor
Relative expression
Relative mRNA expression
Transcript per million
p<0.001
p=0.53
p<0.00001
p=0.05
p=0.014
p=0.0001
p=0.02
p=0.0005
p=0.49
p=0.72
p=0.0099
p<0.0001
p=0.092
p=0.0004
p=0.039
p=0.14
p=0.07
p=0.08
p=0.0009
p=0.06
p=0.01
p=0.002

S4

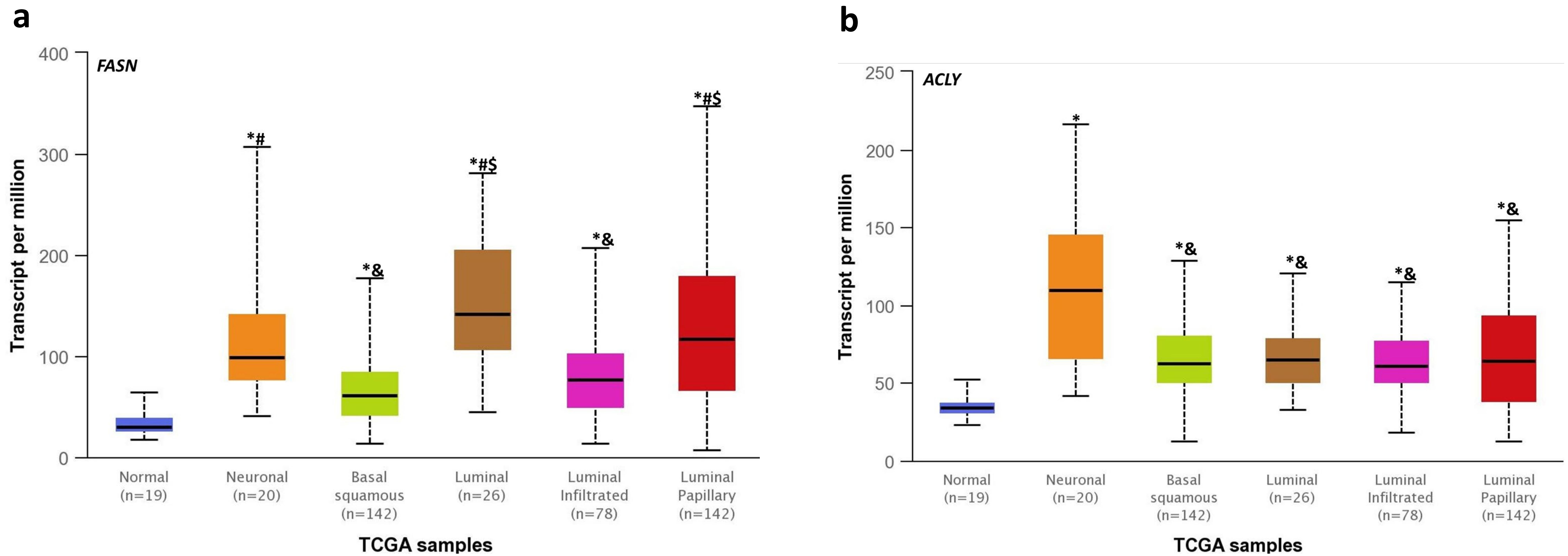

a
FASN
Transcript per million
400
300
200
100
0
*#
*&
*#$
*&
*#$
Normal (n=19)
Neuronal (n=20)
Basal squamous (n=142)
Luminal (n=26)
Luminal Infiltrated (n=78)
Luminal Papillary (n=142)
TCGA samples
b
ACLY
Transcript per million
250
200
150
100
50
0
*
*&
*&
*&
*&
Normal (n=19)
Neuronal (n=20)
Basal squamous (n=142)
Luminal (n=26)
Luminal Infiltrated (n=78)
Luminal Papillary (n=142)
TCGA samples

S5

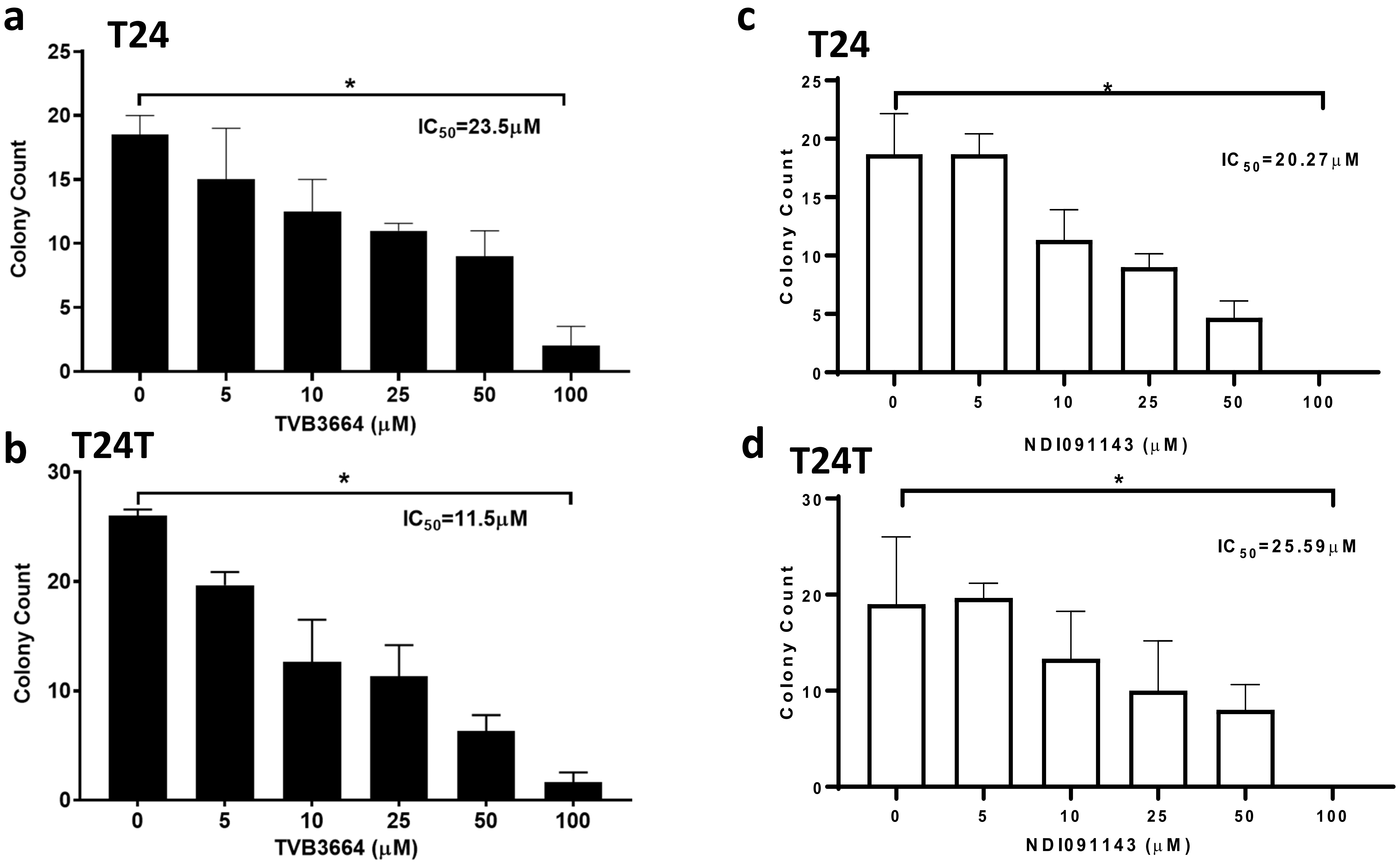

a
T24
Colony Count
IC50=23.5μM
*
0
5
10
25
50
100
TVB3664 (μM)
b
T24T
Colony Count
IC50=11.5μM
*
0
5
10
25
50
100
TVB3664 (μM)
c
T24
Colony Count
IC 50=20.27μM
*
0
5
10
25
50
100
NDI091143 (μM)
d
T24T
Colony Count
IC 50=25.59μM
*
0
5
10
25
50
100
NDI091143 (μM)

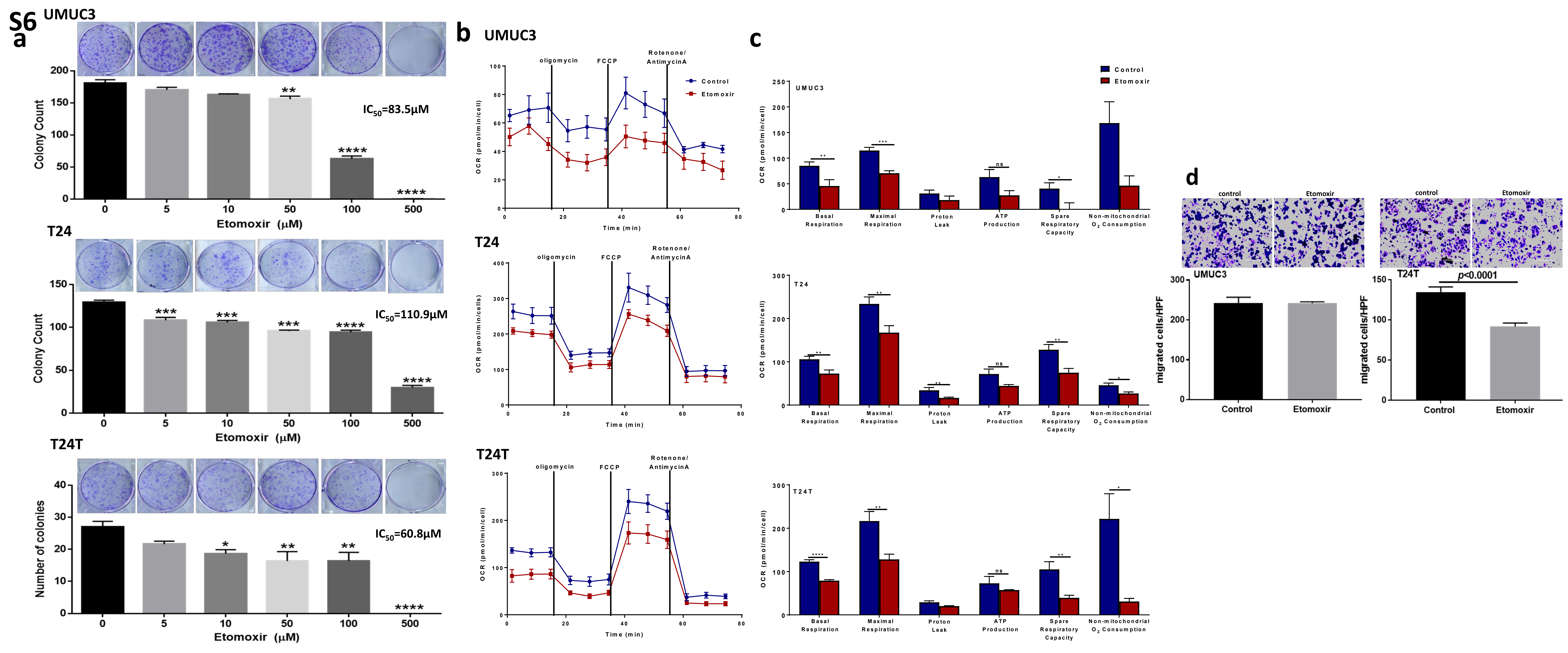

S6
a
UMUC3
Colony Count
IC50=83.5µM
Etomoxir (µM)
T24
IC50=110.9µM
T24T
Number of colonies
IC50=60.8µM
b
UMUC3
oligomycin
FCCP
Rotenone/ AntimycinA
Control
Etomoxir
OCR (pmol/min/cell)
Time (min)
T24
T24T
c
UMUC3
T24
T24T
Basal Respiration
Maximal Respiration
Proton Leak
ATP Production
Spare Respiratory Capacity
Non-mitochondrial O2 Consumption
d
control
Etomoxir
UMUC3
T24T
p<0.0001
migrated cells/HPF
Control
Etomoxir

S7

a

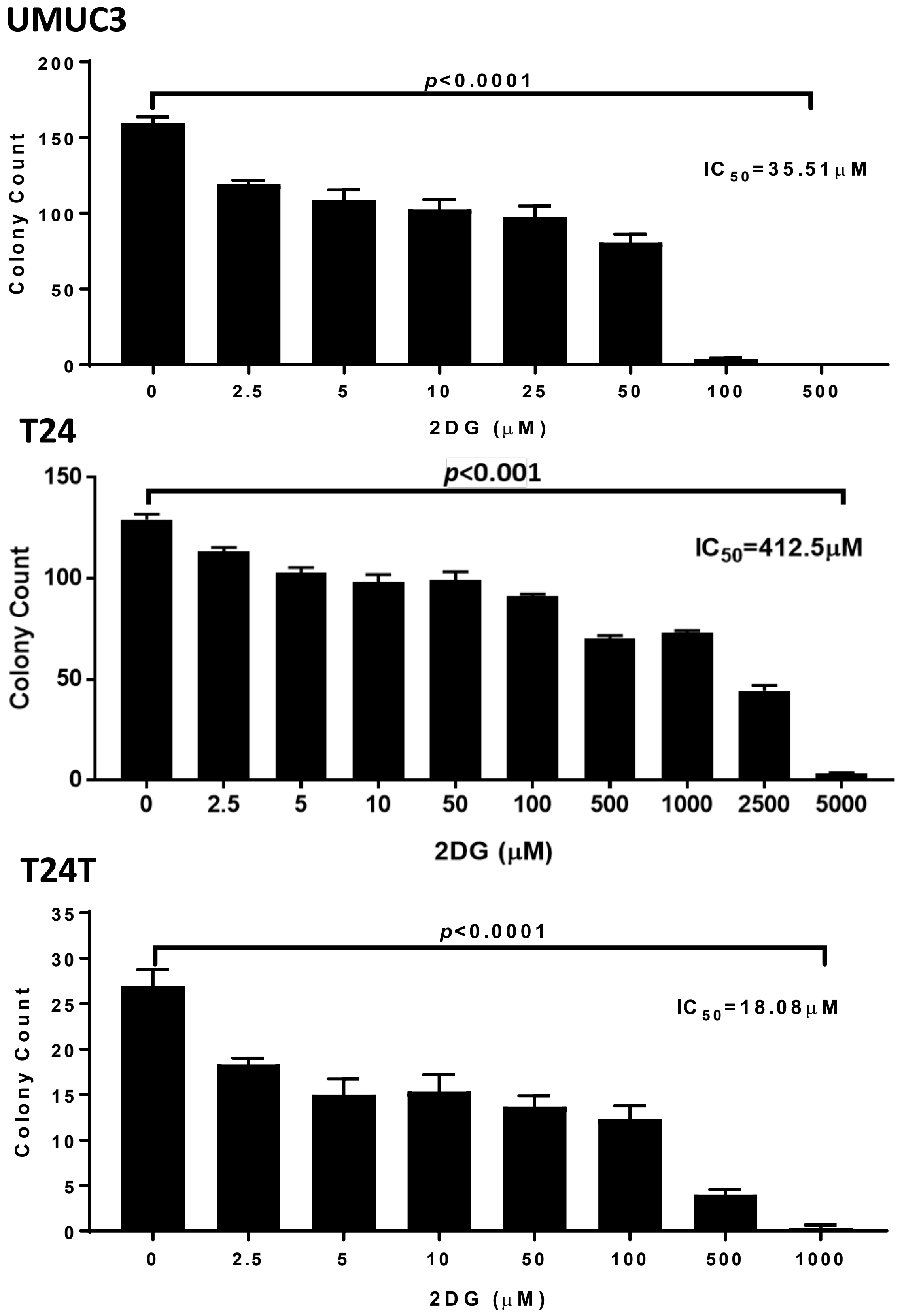
UMUC3
Colony Count
p<0.0001
IC50=35.51μM
0 2.5 5 10 25 50 100 500
2DG (μM)
T24
Colony Count
p<0.001
IC50=412.5μM
0 2.5 5 10 50 100 500 1000 2500 5000
2DG (μM)
T24T
Colony Count
p<0.0001
IC50=18.08μM
0 2.5 5 10 50 100 500 1000
2DG (μM)

b

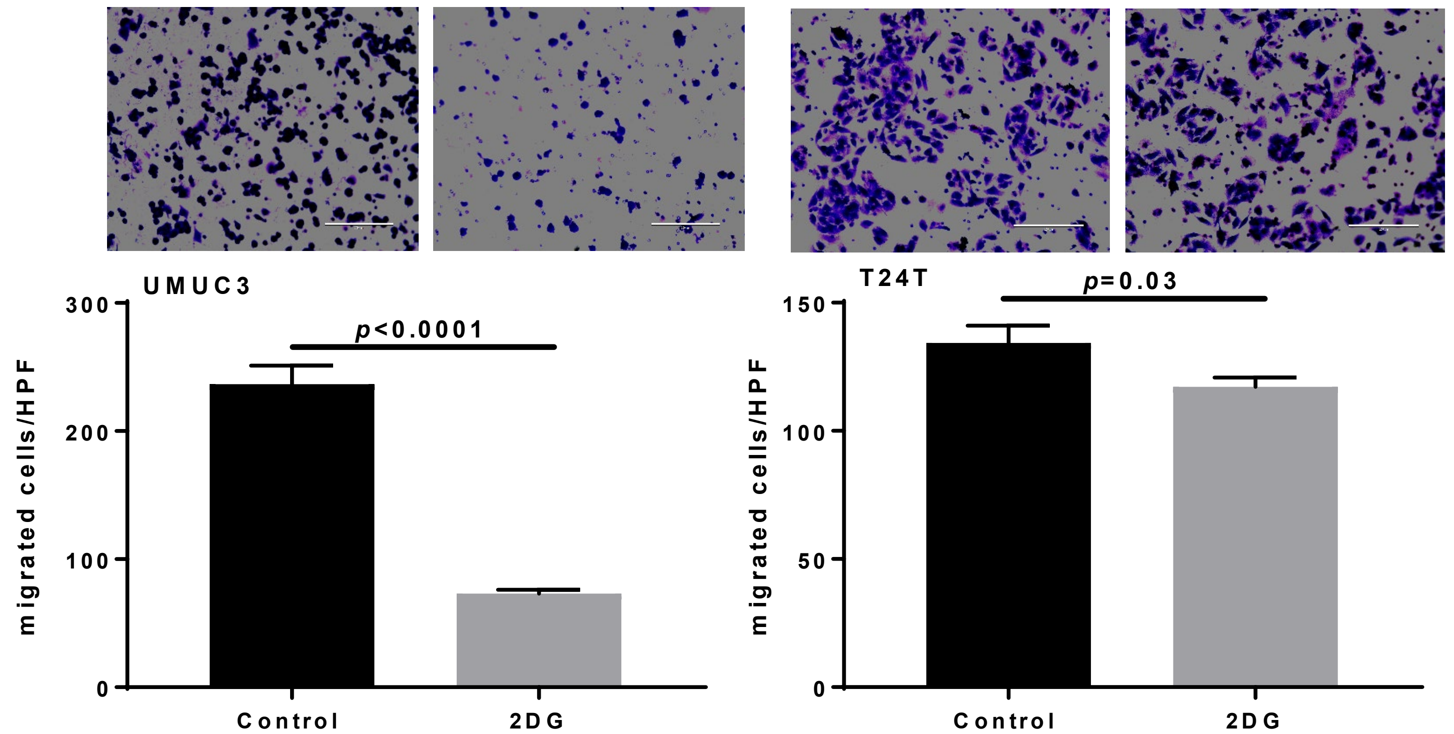
UMUC3
migrated cells/HPF
p<0.0001
Control
2DG
T24T
migrated cells/HPF
p=0.03
Control
2DG

S8

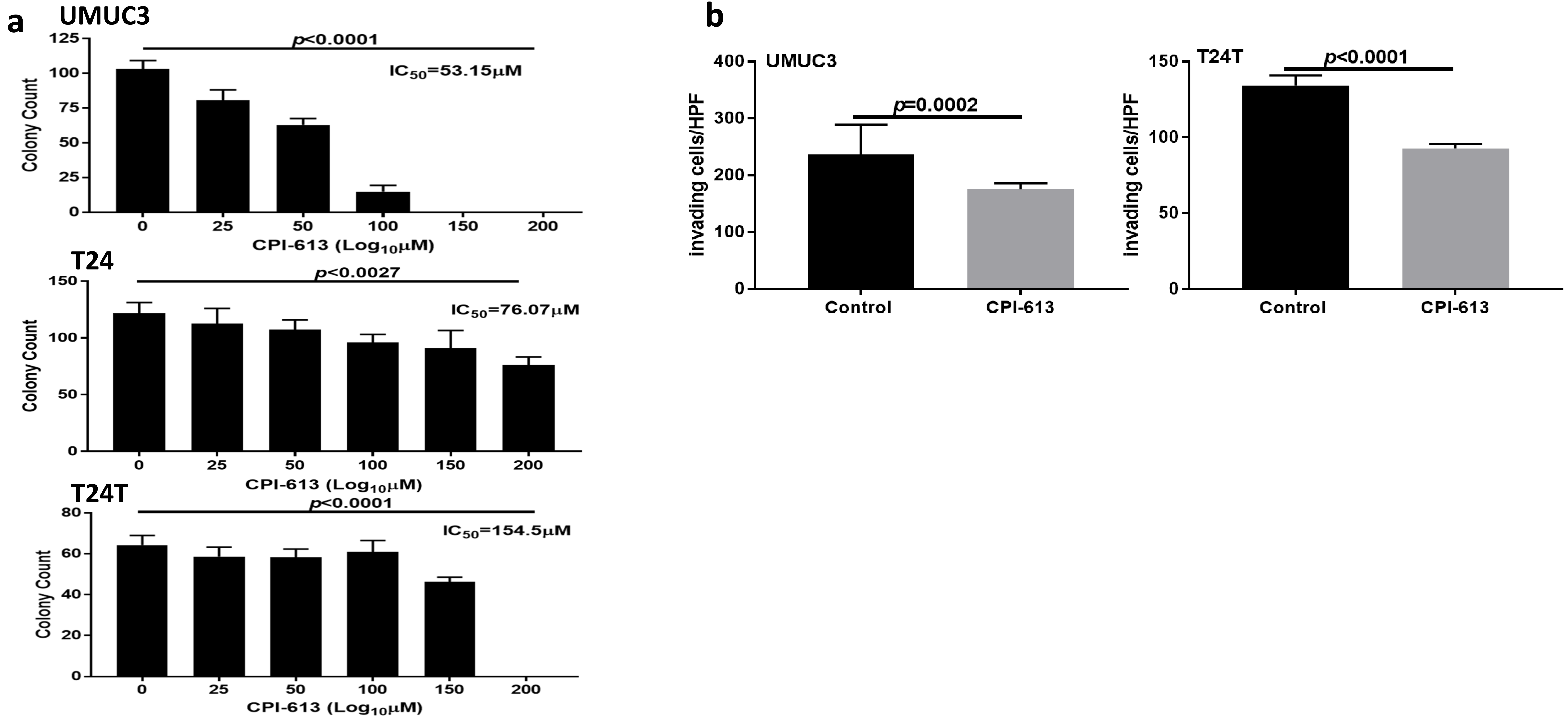
a
UMUC3
p<0.0001
IC50=53.15μM
Colony Count
CPI-613 (Log10μM)
0
25
50
100
150
200
T24
p<0.0027
IC50=76.07μM
T24T
p<0.0001
IC50=154.5μM
b
UMUC3
p=0.0002
invading cells/HPF
Control
CPI-613
T24T
p<0.0001

S9

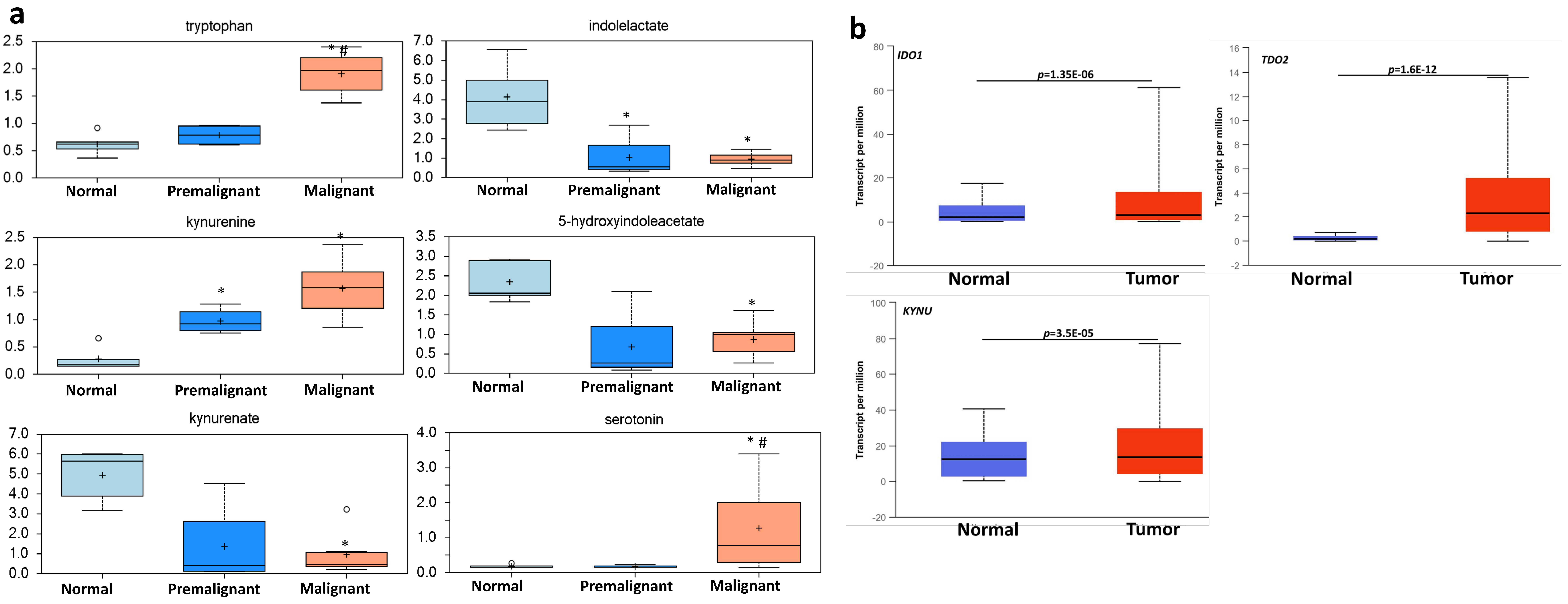
a
tryptophan
indolelactate
kynurenine
5-hydroxyindoleacetate
kynurenate
serotonin
Normal
Premalignant
Malignant
* #
*
b
IDO1
p=1.35E-06
TDO2
p=1.6E-12
KYNU
p=3.5E-05
Transcript per million
Normal
Tumor

S10

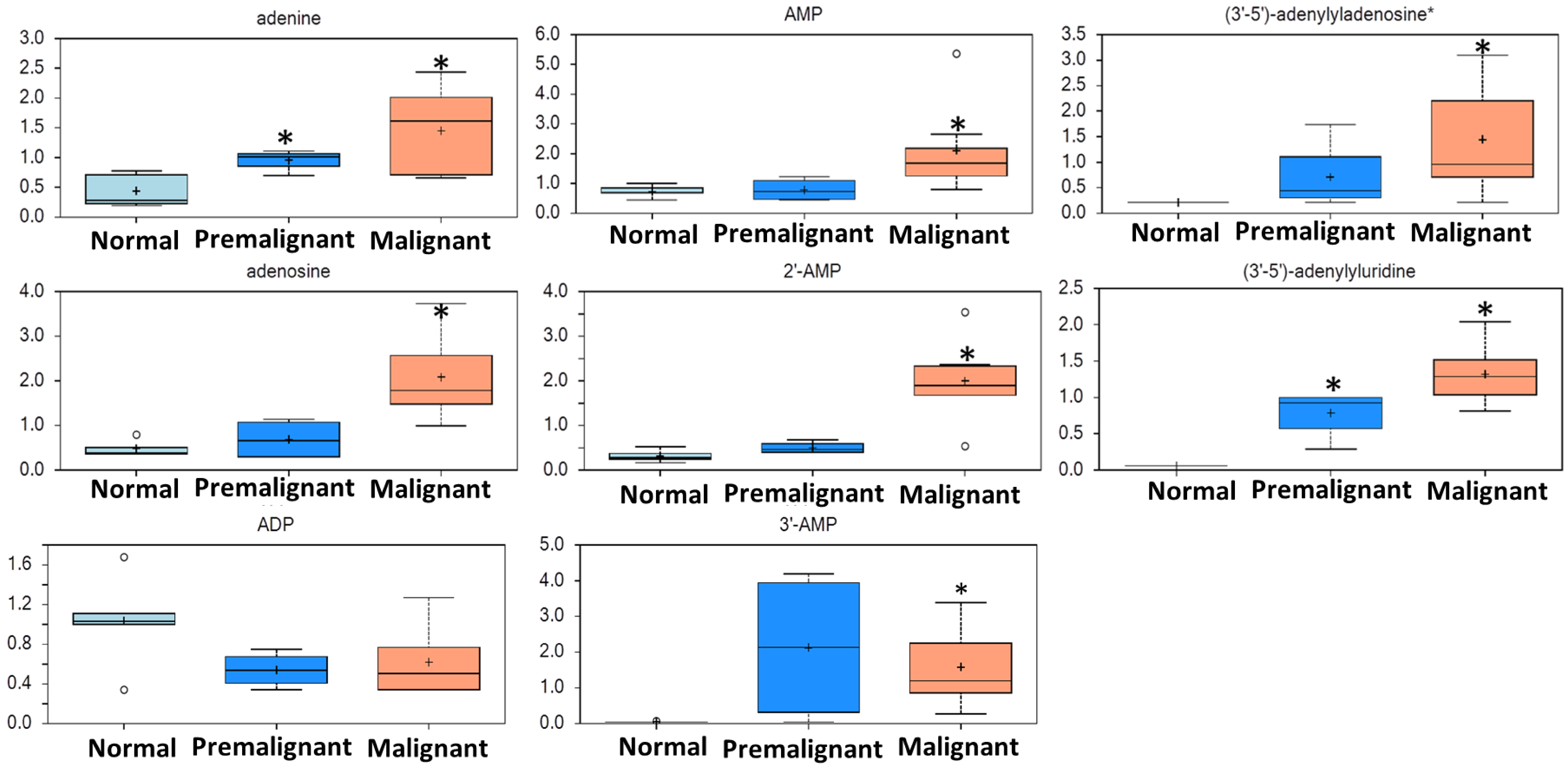

adenine
3.0
2.5
2.0
1.5
1.0
0.5
0.0
Normal
Premalignant
Malignant
AMP
6.0
5.0
4.0
3.0
2.0
1.0
0.0
(3'-5')-adenylyladenosine*
3.5
adenosine
2'-AMP
(3'-5')-adenylyluridine
ADP
1.6
1.2
0.8
0.4
3'-AMP

Supplement Figure Legends:

Supplement Figure 1: Expression of the transcripts of lipases and phospholipases in patients' tumors is significantly upregulated in bladder tumors compared to normal. p-values were determined by Student's t-test.

Supplement Figure 2: The expression levels of eicosanoids prostaglandins E2 and F2a as well as 12-HETE and 15-HETE in *TCGA, GSE13507, MSKCC, and GSE3167. p*-values were determined by Student's *t*-test.

Supplement Figure 3: Expression of enzymes involved in **a.** *de novo* lipogenesis, and **b.** Fatty acid oxidation (FAO) in *TCGA, GSE13507, MSKCC, and GSE3167. p*-values were determined by Student's *t*-test.

Supplement Figure 4: a. The expression of *FASN* transcript in different molecular subtypes of BCa. **b.** The expression of *ACLY* transcript in different molecular subtypes of BCa. $p<0.01$ *compared to normal; # compared to basal squamous, $ compared to luminal infiltrated, &, compared to neuronal.

Supplement Figure 5: a-b. FASN inhibitor TVB3664, and **c-d.** ACLY inhibitor NDI091143 exerts dose-dependent inhibition of T24 and T24T clonogenic survival. *$p<0.05$, one way ANOVA.

Supplement Figure 6: Effects of inhibition of CPT1a by etomoxir on malignant phenotype of BCa cells: a. Etomoxir exerts dose-dependent inhibition of clonogenic survival of UMUC3, T24 and T24T: upper photomicrographs of BCa cell colonies. Bar graphs represent means±SEM of a representative experiment that was performed in triplicates. **b.** Line graphs of the real-time tracing of oxygen consumption rate (OCR) and mito-stress test in BCa cells treated with etomoxir. **c.** Bar graphs represent means±SEM of indicated measurements (n=5/experimental condition) repeated twice. **d.** effect of etomoxir on UMUC3 and T24T matrix invasion. Bars report mean±SEM of number of invading cells/high power field (HPF). *$p<0.05$, **$p<0.01$, ***$p<0.001$, ****$p<0\text{-}0001$ and ns, not significant as determined by one way ANOVA (**a**) and Students' *t*-test (**c-d**).

Supplement Figure 7: a. 2-DG exerts dose-dependent inhibition of clonogenic survival of UMUC3, T24 and T24T. Bar graphs represent means±SEM of the number of colonies/well. representative of 2 experiment each performed in triplicates. *p*-values were determined by One way ANOVA. **b.** 2DG inhibits UMUC3 and T24T invasion. Bars report mean±SEM of number of invading cells/high power field (HPF). *p*-values were determined by Students' *t*-test.

Supplement Figure 8: a. CPI-613 exerts dose-dependent inhibition of clonogenic survival of UMUC3, T24 and T24T. Bar graphs represent means±SEM of the number of colonies/well. representative of 2 experiment each performed in triplicates. *p*-values were determined by one way ANOVA. **b.** CPI-613 inhibits UMUC3 and T24T invasion. Bars, report mean±SEM of number of invading cells/high power field (HPF). *p*-values were determined by Students' *t*-test.

Supplement Figure 9: Tryptophan metabolism: a. Box plots show the increase in tryptophan and kynurenine in premalignant and malignant murine bladder lesions together with decrease in their degradation products. *$p<0.05$ compared to normal bladders (n=5). #$p<0.05$ comparing premalignant lesion (n=4) to early malignant/NMIBC (n=7). *p*-values were determined by one way ANOVA with multiple comparisons. **b.** The expression of the key enzymes involved in tryptophan metabolism in TCGA data. *p*-values were determined by Student's *t*-test.

Supplement Figure 10: Increased adenine-containing purine metabolites in murine bladder lesions. *$p<0.05$ compared to normal bladders (n=5). #$p<0.05$ comparing premalignant lesion (n=4) to early malignant/NMIBC (n=7). *p*-values were determined by one way ANOVA with multiple comparisons.
